# Normal Gestational Weight Gain Protects From Large-for-Gestational-Age Birth Among Women With Obesity and Gestational Diabetes

**DOI:** 10.3389/fpubh.2021.550860

**Published:** 2021-05-31

**Authors:** Sanna Mustaniemi, Hilkka Nikkinen, Aini Bloigu, Anneli Pouta, Risto Kaaja, Johan G. Eriksson, Hannele Laivuori, Mika Gissler, Eero Kajantie, Marja Vääräsmäki

**Affiliations:** ^1^PEDEGO Research Unit, Medical Research Center Oulu, Oulu University Hospital, University of Oulu, Oulu, Finland; ^2^Department of Public Health and Welfare, Population Health Unit, Finnish Institute for Health and Welfare, Oulu, Finland; ^3^Department of Government Services, Finnish Institute for Health and Welfare, Helsinki, Finland; ^4^Turku University Hospital, Turku University, Turku, Finland; ^5^Department of General Practice and Primary Health Care, Helsinki University Hospital, University of Helsinki, Helsinki, Finland; ^6^Folkhälsan Research Center, Helsinki, Finland; ^7^Department of Obstetrics and Gynecology, Human Potential Translational Research Programme, Yong Loo Lin School of Medicine, National University of Singapore, Singapore, Singapore; ^8^Department of Obstetrics and Gynecology, Faculty of Medicine and Health Technology, Tampere University Hospital, Tampere University, Tampere, Finland; ^9^Medical and Clinical Genetics, Helsinki University Hospital, University of Helsinki, Helsinki, Finland; ^10^Institute for Molecular Medicine Finland, Helsinki Institute of Life Science, University of Helsinki, Helsinki, Finland; ^11^Information Services Department, Finnish Institute for Health and Welfare, Helsinki, Finland; ^12^Department of Neurobiology, Care Sciences and Society, Karolinska Institute, Stockholm, Sweden; ^13^Children's Hospital, Helsinki University Hospital, University of Helsinki, Helsinki, Finland; ^14^Department of Clinical and Molecular Medicine, Norwegian University of Science and Technology, Trondheim, Norway

**Keywords:** gestational diabetes, gestational weight gain, obesity, birthweight, birth weight SD, large-for-gestational age

## Abstract

**Background:** Pre-pregnancy obesity, excess gestational weight gain (GWG), and gestational diabetes (GDM) increase fetal growth. Our aim was to assess whether normal GWG is associated with lower risk for a large-for-gestational-age (LGA; over the 90th percentile of birth weight for sex and gestational age) infant and lower birth weight standard deviation (SD) score in the presence of GDM and maternal obesity.

**Methods:** This multicenter case-control study is part of the Finnish Gestational Diabetes (FinnGeDi) Study and includes singleton pregnancies of 1,055 women with GDM and 1,032 non-diabetic controls. Women were divided into 12 subgroups according to their GDM status, pre-pregnancy body mass index (BMI; kg/m^2^), and GWG. Non-diabetic women with normal BMI and normal GWG (according to Institute of Medicine recommendations) served as a reference group.

**Results:** The prevalence of LGA birth was 12.2% among women with GDM and 6.2% among non-diabetic women (*p* < 0.001). Among all women, normal GWG was associated with lower odds of LGA [odds ratio (OR) 0.57, 95% CI: 0.41–0.78]. Among women with both obesity and GDM, the odds for giving birth to a LGA infant was 2.25-fold (95% CI: 1.04–4.85) among those with normal GWG and 7.63-fold (95% CI: 4.25–13.7) among those with excess GWG compared with the reference group. Compared with excess GWG, normal GWG was associated with 0.71 SD (95% CI: 0.47–0.97) lower birth weight SD score among women with GDM and obesity. Newborns of normal weight women with GDM and normal GWG had 0.28 SD (95% CI: 0.05–0.51) lower birth weight SD scores compared with their counterparts with excess GWG. In addition, in the group of normal weight non-diabetic women, normal GWG was associated with 0.46 SD (95% CI: 0.30–0.61) lower birth weight SD scores compared with excess GWG.

**Conclusion:** GDM, obesity, and excess GWG are associated with higher risk for LGA infants. Interventions aiming at normal GWG have the potential to lower LGA rate and birth weight SD scores even when GDM and obesity are present.

## Introduction

Gestational diabetes (GDM) affects 10–25% of all pregnancies depending on diagnostic criteria, screening strategies, and study populations (1, 2). Along with GDM, pre-pregnancy obesity and excess gestational weight gain (GWG) are major pregnancy-related health problems. During pregnancy, GWG is an important modifiable factor. Nearly half of all pregnant women exceed the Institute of Medicine (IOM) recommendations for GWG (3, 4).

GDM, pre-pregnancy obesity, and excess GWG are all independent risk factors for having a large-for-gestational-age (LGA) infant (4–7). Previous studies assessing the association of GWG with LGA in relation to GDM and pre-pregnancy obesity are inconsistent. One register-based study reported that higher GWG was associated with higher risk for LGA among both women with untreated GDM and among those without GDM (8). Previous retrospective studies in a general population of full-term singleton births reported that excess GWG was associated with LGA, but the prevalence of GDM in these studies was low ranging from 4.5 to 4.9% (5, 7). Other studies including only women with GDM reported an increased risk for LGA in women with excess GWG (6, 9–11).

Therefore, we studied whether GWG is associated with LGA infants in women with and without GDM as categorized by pre-pregnancy BMI. We hypothesized that normal GWG decreases the risk of LGA in all BMI categories regardless of GDM status.

## Materials and Methods

The present multicenter case-control study is based on the clinical-genetic arm of the Finnish Gestational Diabetes Study (FinnGeDi), which has been described in detail (12, 13). Briefly, 1,146 women with singleton pregnancies diagnosed with GDM were recruited in delivery units before delivery, and the next consenting non-diabetic mother (*n* = 1,066) giving birth in the same hospital was recruited as a control. Women were recruited between February 2009 and December 2012 in seven Finnish delivery hospitals. Comprehensive data on participants, pregnancy, delivery, and newborns were obtained from hospital and maternal welfare clinic records, from a detailed self-filled questionnaire, and from the Finnish Medical Birth Register.

According to the national Finnish Current Care guidelines introduced in 2008, an oral glucose tolerance test (OGTT) is recommended to screen for GDM in every pregnant woman, except those with very low-risk: (1) <25 year-old primiparous women with BMI <25 kg/m^2^ and without a family history of diabetes; and (2) <40 year-old multiparous women with BMI <25 kg/m^2^ and without a history of GDM or a macrosomic newborn (birth weight > 4,500 g) (14). GDM was diagnosed using a 2-hour, 75 g OGTT that was primarily performed between 24 and 28 weeks of gestation. For high-risk groups (prior GDM, BMI ≥ 35 kg/m^2^, glucosuria, family history of diabetes, or polycystic ovary syndrome), OGTT was performed for the first time between 12 and 16 weeks of gestation and, if normal, repeated between 24 and 28 weeks of gestation. The diagnostic criteria for the plasma glucose concentrations were ≥ 5.3 mmol/l after overnight fasting, ≥ 10.0 mmol/l at 1 h, or ≥ 8.6 mmol/l at 2 h after the glucose load. The diagnosis of GDM was based on one or more abnormal values in the OGTT. Additionally, GDM diagnosis was based on glucose self-monitoring for 24 participants (12).

Information on maternal age at delivery, parity, and smoking during pregnancy were obtained from the Finnish Medical Birth Register, educational attainment from a questionnaire, and use of insulin or metformin from the mother's medical records. Self-reported maternal height and weight before pregnancy, and weight measured in the first and last antenatal visit were obtained from maternity welfare clinic records. Pre-pregnancy BMI <25.0 kg/m^2^ was classified as normal, 25.0–29.9 kg/m^2^ as overweight, and ≥ 30.0 kg/m^2^ as obese (15). Underweight women (BMI <18.5 kg/m^2^) (*n* = 58, 2.8%) were categorized as normal in the analysis. GWG was calculated as the difference between pre-pregnancy weight and weight at the last antenatal visit. The 2009 IOM recommendations were used to classify normal and excess weight gain during pregnancy in different pre-pregnancy BMI categories (normal: 11.5–16.0 kg; overweight: 7.0–11.5 kg; obese: 5.0–9.0 kg)^11^. Those women with GWG below the IOM recommendations (n = 434, 20.8%) were classified as having normal GWG in the final analysis.

Data on birth weight (kg), birth length (cm), head circumference (cm), and sex of the newborn were obtained from the Finnish Medical Birth Register. The birth weight standard deviation (SD) score is a sex- and parity-specific parameter estimating birth weight and length in singletons born at 23–43 gestational weeks, according to Finnish standards (16). LGA was defined as birth weight over the 90th percentile and small-for-gestational-age (SGA) was defined as under the 10th percentile for sex and gestational age.

**Figure 1:**
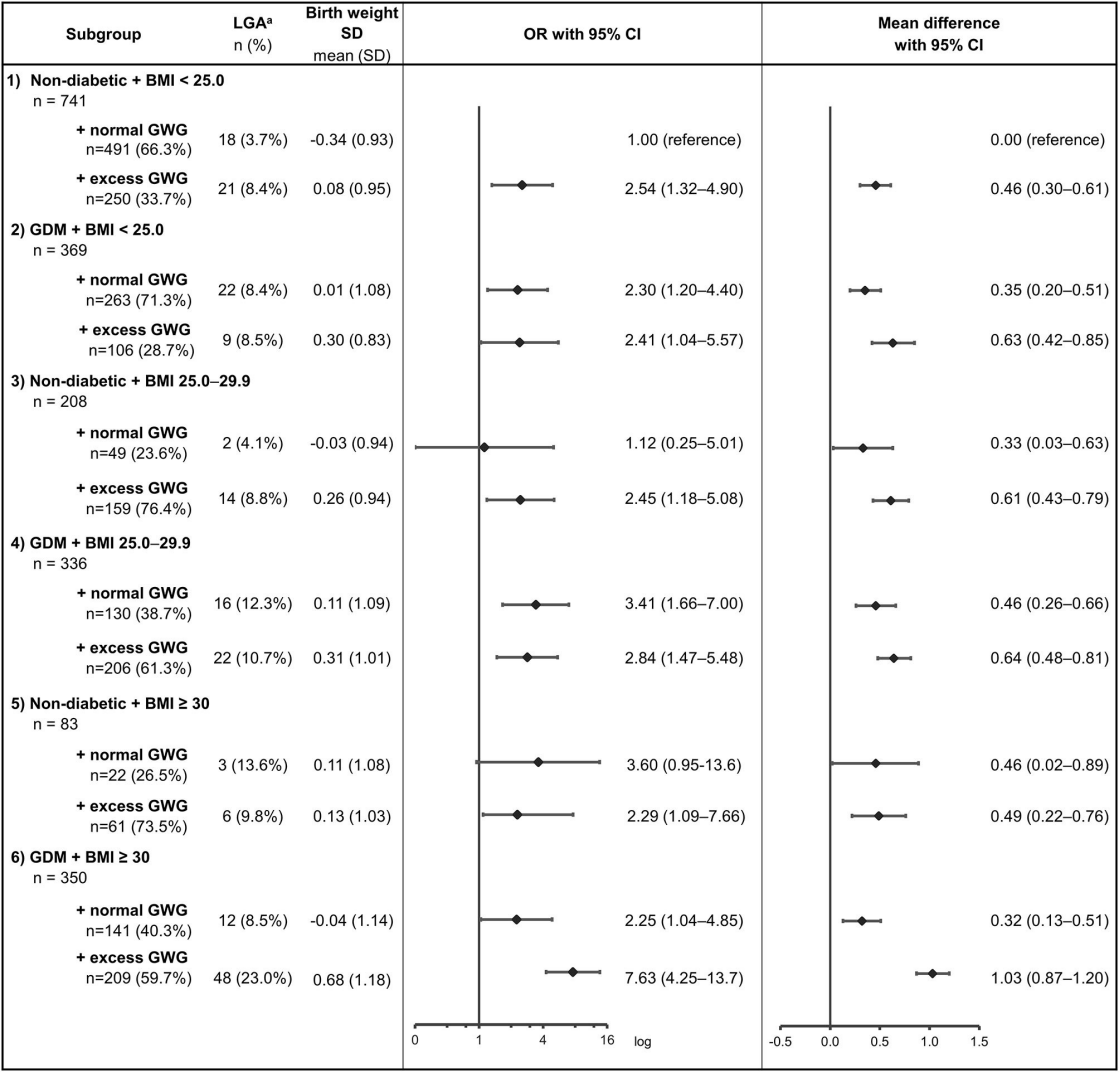
Odds ratios (ORs) for large-for-gestational age (LGA) and mean differences for birthweight standard deviation (SD) scores in 12 subgroups categorized by GDM status, pre-pregnancy BMI, and GWG (normal or excess); the group of non-diabetic women with normal pre-pregnancy BMI and with normal GWG was used as a reference group. GDM, gestational diabetes; BMI, body mass index (kg/m^2^); GWG, gestational weight gain. Linear regression for continuous variables and logistic regression for categorical variables adjusted for participant's age at delivery, parity, smoking during pregnancy, and delivery hospital.

Of all the 2,212 participants, 47 (2.1%) women with missing GWG data and 78 (3.5%) women whose last antenatal weight was measured before 35 weeks were excluded from the analysis. In total, 1,055 women with GDM and 1,032 non-diabetic controls were included in the analysis (Table 1). The characteristics of women and their newborns were compared in six groups categorized by GDM status (case or control) and pre-pregnancy BMI (normal, overweight, obese) (Tables 2, 3). Further, participants were divided into 12 subgroups according to their GDM status, pre-pregnancy BMI, and GWG (normal or excess) (Figure 1). The group of non-diabetic controls with normal pre-pregnancy BMI and with normal GWG was used as a reference group. The study was approved by the Ethics Committee in Northern Ostrobothnia Hospital District in 2008. Each participant provided written informed consent.

**Table 1:** Characteristics of mothers and newborns (*n* = 2,087).

**Characteristic**	**Non-diabetic** ***n* = 1,032**	**GDM** ***n* = 1,055**	***P*-value^a^**
	**Mean (SD)/*****n*** **(%)**		
**Mother**			
Age at delivery, years	29.5 (5.1)	32.0 (5.3)	<0.001
Height, cm	165.4 (5.8)	164.8 (5.7)	0.029
Pre-pregnancy weight, kg	64.6 (12.0)	76.5 (17.2)	<0.001
Pre-pregnancy BMI, kg/m^2^	23.6 (4.1)	28.1 (6.1)	<0.001
Smoking during pregnancy, n (%)	153 (14.8%) (1,031)	178 (16.9%) (1,051)	0.191
Education, (%)	(908)	(948)	
Basic or less	40 (4.4%)	61 (6.4%)	0.054
Secondary	410 (45.2%)	438 (46.2%)	0.650
Lower-level tertiary	229 (25.2%)	255 (26.9%)	0.410
Upper-level tertiary	229 (25.2%)	194 (20.5%)	0.015
Primipara, *n* (%)	505 (48.9%)	455 (43.1%)	0.008
GWG, kg^b^	14.8 (5.1)	12.3 (5.8)	<0.001
Excess GWG^c^, *n* (%)	470 (45.5%)	521 (49.4%)	0.079
Insulin and/or metformin treatment	–(0.0%)	190 (18.0%)	<0.001
**Newborn**			
Birth weight, g	3,591 (471)	3,670 (477)	<0.001
Birth weight SD	−0.10 (0.98)	0.24 (1.10)	<0.001
SGA^d^, *n* (%)	111 (10.8%)	94 (8.9%)	0.157
LGA^e^, *n* (%)	64 (6.2%)	129 (12.2%)	<0.001
Gestational age at birth, weeks	40.2 (1.2)	39.7 (1.2)	<0.001

*GDM, gestational diabetes; BMI, body mass index (kg/m^2^); GWG, gestational weight gain*.

*() Number of available information unless from all*.

a *The t-test for continuous and chi-square test for categorical variables*.

b *Difference of (self-reported) pre-pregnancy weight and weight at the last antenatal visit at 35 gestational weeks or later*.

c *Excess gestational weight gain based on Institute of Medicine 2009 criteria*.

d *Small-for-gestational-age (birth weight under the 10th percentile for sex and gestational age)*.

e *Large-for-gestational-age (birth weight over the 90th percentile for sex and gestational age)*.

**Table 2:** Characteristics of mothers and newborns in six groups categorized by GDM status and pre-pregnancy BMI (*n* = 2,087).

**Number of subgroup**	**1**	**2**	**3**	**4**	**5**	**6**
	**Non-diabetic +** **BMI <25.0** ***n* = 741**	**GDM +** **BMI <25.0** ***n* = 369**	**Non-diabetic +** **BMI 25.0–29.9** ***n* = 208**	**GDM +** **BMI 25.0–29.9** ***n* = 336**	**Non-diabetic +** **BMI ≥ 30.0** ***n* = 83**	**GDM +** **BMI ≥ 30.0** ***n* = 350**
	**Mean (SD)/*****n*** **(%)**					
**Mother**						
Age at delivery, years	29.3 (5.1)	31.7 (5.2)	30.5 (5.2)	32.4 (5.3)	29.1 (5.2)	31.8 (5.4)
Height, cm	165.4 (5.8)	165.0 (5.9)	165.4 (6.3)	164.7 (5.7)	165.1 (5.4)	164.7 (5.7)
Pre-pregnancy weight, kg	59.1 (6.8)	60.8 (6.8)	73.7 (6.5)	73.8 (6.6)	90.9 (10.3)	95.5 (13.4)
Pre-pregnancy BMI, kg/m^2^	21.6 (2.0)	22.3 (1.9)	26.9 (1.4)	27.2 (1.4)	33.3 (3.2)	35.2 (4.4)
Smoking during pregnancy, *n* (%)^a^	96 (13.0%)	45 (12.2%)	38 (18.3%)	49 (14.6%)	19 (22.9%)	84 (24.1%)
**Education, (%)^b^**						
Basic or less	27 (4.2%)	12 (3.6%)	8 (4.2%)	25 (8.1%)	5 (7.0%)	24 (7.8%)
Secondary	284 (43.8%)	123 (37.0%)	87 (46.0%)	139 (45.0%)	39 (54.9%)	176 (57.3%)
Lower-level tertiary	149 (23.0%)	93 (28.0%)	61 (32.3%)	88 (28.5%)	19 (26.8%)	74 (24.1%)
Upper-level tertiary	188 (29.0%)	104 (31.3%)	33 (17.5%)	57 (18.4%)	8 (11.3%)	33 (10.7%)
Primipara, *n* (%)	386 (52.1%)	180 (48.8%)	86 (41.3%)	131 (39.0%)	33 (39.8%)	144 (41.1%)
GWG, kg^c^	14.8 (4.6)	14.0 (5.1)	15.2 (5.5)	13.1 (5.4)	13.7 (7.5)	9.9 (6.0)
Excess GWG^d^, *n* (%)	250 (33.7%)	106 (28.7%)	159 (76.4%)	206 (61.3%)	61 (73.5%)	209 (59.7%)
GWG below recommended^e^, *n* (%)	184 (24.8%)	119 (32.2%)	13 (6.3%)	38 (11.3%)	8 (9.6%)	72 (20.6%)
**Newborn**						
Birth weight, g	3,531 (461)	3,610 (469)	3,741 (451)	3,677 (469)	3,745 (495)	3,726 (486)
Birth weight SD	−0.20 (0.96)	0.09 (1.02)	0.19 (0.94)	0.23 (1.04)	0.12 (1.03)	0.39 (1.22)
SGA^f^, *n* (%)	95 (12.8%)	38 (10.3%)	10 (4.8%)	28 (8.3%)	6 (7.2%)	28 (8.0%)
LGA^g^, *n* (%)	39 (5.3%)	31 (8.4%)	16 (7.7%)	38 (11.3%)	9 (10.8%)	60 (17.1%)
Gestational age at birth, weeks	40.1 (1.2)	39.8 (1.2)	40.3 (1.2)	39.8 (1.2)	40.5 (1.1)	39.7 (1.3)

*GDM, gestational diabetes; BMI, body mass index (kg/m^2^); GWG, gestational weight gain*.

a *Number of missing data in order of subgroups 1/1/0/1/0/2*.

b *Number of missing data in order of subgroups 93/37/19/27/12/43*.

c *Difference of (self-reported) pre-pregnancy weight and weight at the last antenatal visit at 35 gestational weeks or later*.

d *Excess gestational weight gain based on the Institute of Medicine 2009 criteria*.

e *Gestational weight gain below the Institute of Medicine 2009 criteria*.

f *Small-for-gestational-age (birth weight under the 10th percentile for sex and gestational age)*.

g *Large-for-gestational-age (birth weight over the 90th percentile for sex and gestational age)*.

**Table 3:** Adjusted^a^ odds ratios (ORs) and mean differences (95% CI) of mother's and newborn's characteristics as divided into five subgroups (2–6) compared with normal weight non-diabetic women (subgroup 1).

**Number of subgroup**	**2**	**3**	**4**	**5**	**6**
	**GDM** **+** **BMI** **<** **25.0** ***n*** **=** **369**	**Non-diabetic** **+** **BMI 25.0–29.9** ***n*** **=** **208**	**GDM** **+** **BMI 25.0–29.9** ***n*** **=** **336**	**Non-diabetic** **+** **BMI** **≥** **30.0** ***n*** **=** **83**	**GDM** **+** **BMI** **≥** **30.0** ***n*** **=** **350**
	**OR/mean difference (95% CI)^a^**				
**Mother**					
Age at delivery, years	2.3 (1.7–2.9)	1.2 (0.5–1.9)	2.7 (2.1–3.3)	−0.1 (−1.2 to 0.9)	2.5 (1.9–3.1)
Height, cm	−0.7 (−1.4 to 0.1)	0.08 (−0.8 to 1.0)	−0.9 (−1.7 to −0.2)	−0.2 (−1.5 to 1.1)	−0.7 (−1.5 to 0.1)
Smoking during pregnancy	1.27 (0.86–1.88)	1.72 (1.12–2.64)	1.68 (1.14–2.48)	1.92 (1.08–3.42)	2.94 (2.08–4.17)
Primipara	1.17 (0.90–1.53)	0.74 (0.53–1.03)	0.83 (0.63–1.10)	0.58 (0.36–0.94)	0.89 (0.67–1.17)
GWG, kg^b^	−0.5 (−1.2 to 0.1)	0.6 (−0.2 to 1.4)	−1.2 (−1.9 to −0.6)	−1.1 (−2.3 to 0.1)	−4.5 (−5.2 to −3.9)
Excess GWG^c^	0.87 (0.66–1.16)	7.38 (5.13–10.6)	3.82 (2.88–5.07)	5.95 (3.52–10.0)	3.45 (2.61–4.56)
GWG below recommended^d^	1.33 (1.00–1.76)	0.19 (0.11–0.34)	0.33 (0.23–0.49)	0.32 (0.15–0.68)	0.71 (0.51–0.98)
**Newborn**					
Birth weight, g	69 (10–128)	194 (123–265)	123 (62–184)	201 (95–306)	183 (122–243)
Birth weight SD	0.29 (0.16–0.42)	0.40 (0.24–0.55)	0.43 (0.29–0.57)	0.33 (0.10–0.56)	0.60 (0.46–0.73)
SGA^e^	0.70 (0.46–1.05)	0.31 (0.16–0.61)	0.52 (0.33–0.82)	0.49 (0.21–1.16)	0.48 (0.31–0.76)
LGA^f^	1.59 (0.97–2.61)	1.48 (0.81–2.71)	2.09 (1.30–3.34)	2.14 (0.99–4.62)	3.56 (2.29–5.52)
Gestational age at birth, weeks	−0.3 (−0.5 to −0.2)	0.2 (0.01–0.4)	−0.3 (−0.5 to −0.2)	0.4 (0.1–0.7)	−0.4 (−0.6 to −0.3)

*GDM, gestational diabetes; BMI, body mass index (kg/m^2^); GWG, gestational weight gain*.

a *Linear regression for continuous variables and logistic regression for categorical variables adjusted for participant's age at delivery, parity, smoking during pregnancy, and delivery hospital*.

b *Difference of (self-reported) pre-pregnancy weight and weight at the last antenatal visit at 35 gestational weeks or later*.

c *Excess gestational weight gain based on the Institute of Medicine 2009 criteria*.

d *Gestational weight gain below the Institute of Medicine 2009 criteria*.

e *Small-for-gestational-age (birth weight under the 10th percentile for sex and gestational age)*.

f *Large-for-gestational-age (birth weight over the 90th percentile for sex and gestational age)*.

### Statistical Analysis

Numbers and frequencies (%) are reported for categorical variables and means (SD) for continuous variables. Linear regression [difference in means with 95% confidence interval (CI)] was used for continuous outcomes, and logistic regression [odds ratios (ORs) with 95% CI] was used for categorical outcomes. In the regression analyses, 11 subgroups categorized by GDM status, BMI, and GWG (normal/excess) were compared with the reference group (normal weight non-diabetic women with normal GWG). The results were adjusted for age at delivery, parity, smoking during pregnancy, and delivery hospital. Educational attainment, which is a potential confounding variable, was missing for 11.1% (*n* = 231) of the women; thus, we performed a sub-analysis that included only the women with valid education data. Since adjustment for educational attainment did not essentially alter the associations, it was excluded from the final analysis.

## Results

### Characteristics of Women With and Without GDM

Women with GDM tended to be older, shorter, and had higher BMI than the non-diabetic women (Table 1). Eighty-two percent (*n* = 865) of them were treated with diet, and 18% (*n* = 190) with additional insulin and/or metformin. Of all GDM women, 65.0% were overweight or obese, the corresponding rate being 28.2% among non-diabetic women.

GDM women gained less weight during pregnancy than non-diabetic women (Table 1). Among women with overweight, the difference was 1.8 kg (*p* < 0.001), and among women with obesity, the difference was 3.4 kg (*p* < 0.001) (Table 3). Still, GWG was normal in 50.6% of the women in the GDM group and in 54.4% of the women in the non-diabetic group (Table 1). Of women with overweight or obesity, 60.5% in the GDM group and 75.0% in the non-diabetic group exceeded IOM recommendations for GWG (Table 2). In women with overweight or obesity, GWG below the IOM guidelines was more common in the GDM group, 16.0 vs. 7.2%, respectively (*p* < 0.001). Of the women with GDM, the prevalence of excess GWG was 51.0% in the diet-treated women and 42.1% among women who received additional medical treatment (*p* = 0.027).

### Gestational Weight Gain and Fetal Growth

#### Large-for-Gestational Age

The prevalence of LGA was 12.2% among GDM women and 6.2% among non-diabetic women (*p* < 0.001) (Table 1). Among women with GDM, the prevalence of LGA was 10.8% in the diet-treated group and 18.9% among women who received additional medical treatment (*p* < 0.001). Among all women, pre-pregnancy obesity increased the risk of LGA 1.75-fold (Table 4). In addition to GDM and pre-pregnancy obesity, other independent risk factors for LGA were a previous macrosomic newborn and excess GWG.

**Table 4:** Crude and adjusted odds ratios (ORs) for risk factors of large-for-gestational-age (LGA) (*n* = 2,087).

**Risk factor**	**Crude OR**	**Adjusted OR^a^**
GDM	2.11 (1.54–2.88)	1.59 (1.12–2.26)
Excess gestational weight gain^b^	1.93 (1.42–2.62)	1.77 (1.29–2.43)
Pre-pregnancy BMI 25.0–29.9 kg/m^2^	1.11 (0.80–1.55)	0.81 (0.57–1.15)
Pre-pregnancy BMI ≥ 30.0 kg/m^2^	2.35 (1.71–3.22)	1.75 (1.24–2.47)
Age ≥ 35 years	1.65 (1.19–2.29)	1.03 (0.99–1.06)
Previous macrosomic newborn > 4,500 g	6.76 (3.97–11.5)	7.31 (3.93–13.6)
GDM treated with insulin and/or metformin	1.94 (1.27–2.96)	1.87 (1.18–2.96)

*LGA, large-for-gestational-age (birth weight over the 90th percentile for sex and gestational age); GDM, gestational diabetes; BMI, body mass index (kg/m^2^)*.

a *All adjusted models include participant's age at delivery, parity, smoking during pregnancy, delivery hospital, pre-pregnancy BMI, GDM, and GWG. As covariates, these variables are entered as continuous except categorical variables: GDM, smoking during pregnancy, and delivery hospital*.

b *Excess gestational weight gain based on the Institute of Medicine 2009 criteria*.

Among all women, those with excess GWG had a 1.77-fold odds for a LGA infant compared with women with normal GWG (Table 4). Of the women with both GDM and obesity, the odds for a LGA infant was 2.25-fold among those with normal GWG and 7.63-fold among those with excess GWG compared with the reference group (*p* < 0.001) (Figure 1).

Among normal weight and overweight GDM women, GWG (normal/excess) was unrelated to the prevalence of LGA (Figure 1). Among non-diabetic women, those with normal pre-pregnancy BMI and normal GWG showed lower risk for LGA than women with normal pre-pregnancy BMI and excess GWG (*p* = 0.006). The results remained similar regardless of whether the women with GWG below IOM recommendations were included in the normal GWG group or observed separately.

#### Birth Weight SD Scores

We assessed birth weight SD scores as continuous variables. In general, they were lower when GWG was within the normal range compared with excess GWG (Figure 1). Newborns of women with GDM, obesity, and normal GWG had 0.71 SD (95% CI: 0.47–0.97) lower birth weight SD score than newborns of women with GDM, obesity, and excess GWG. Newborns of normal weight women with GDM and normal GWG had 0.28 SD (95% CI: 0.05–0.51) lower birth weight SD score than newborns of normal weight women with GDM and excess GWG. In addition, newborns of normal weight non-diabetic women with normal GWG had 0.46 SD (95% CI: 0.30–0.61) lower birth weight SD score compared with their counterparts with excess GWG. Among women with GDM and overweight and among non-diabetic women with overweight or obesity, these differences were not statistically significant.

#### Small-for-Gestational Age

Among all women, GWG below the IOM recommendations increased the odds for SGA 1.62-fold (95% CI: 1.14–2.31) compared with women with normal GWG. The prevalence of SGA was highest (12.8%) in the normal weight non-diabetic women (Table 2). If their GWG was below the IOM recommendation, the risk for SGA was 2.26-fold (95% CI: 1.37–3.75) compared with their counterparts with normal GWG. In the other groups, the increased risk for SGA was not seen in women whose GWG was under the IOM recommendations (data not shown).

## Discussion

We found that normal GWG seems to protect from LGA and lowers birth weight SD scores especially among the high-risk women with GDM and pre-pregnancy obesity, but also among non-diabetic women with normal weight. Excess GWG, GDM, pre-pregnancy obesity, and a previous macrosomic newborn were all independent risk factors for LGA.

The protective effect of normal GWG that we observed was substantial. Among women with GDM and obesity, the prevalence of LGA was more than halved when GWG was normal compared with excess GWG (23.0 vs. 8.5%). All women in the study cohort received lifestyle counseling after GDM diagnosis. The lower GWG among GDM women with overweight/obesity compared with their non-diabetic counterparts could reflect the efficacy of diet counseling.

The optimal weight gain during pregnancy in women diagnosed with GDM is unknown. A previous study among women with GDM reported that modified, slightly restricted GWG targets did not decrease the rate of LGA (11). That study reported that almost half of the women with obesity had already exceeded their IOM total GWG target by the time of GDM diagnosis. In Finland, the national Current Care Guidelines recommend that among women with obesity, weight should not increase much more after GDM diagnosis (14). Still, excess GWG was very common in women with overweight/obesity regardless of their GDM status. These findings suggest that early recognition of these high-risk women and interventions aiming at maintaining normal GWG may decrease the risk of pregnancy complications as macrosomia. Also, excess GWG often leads to postpartum weight retention, which increases risk for complications in the woman's subsequent pregnancies and her long-term morbidity (3).

We found that insufficient GWG doubled the risk for SGA in normal weight non-diabetic women, which is consistent with a recent meta-analysis (17). However, GWG below the recommendations did not increase the risk for SGA in normal weight women with GDM or women with overweight/obesity regardless of GDM status (data not shown), thus being in line with a previous study (11). However, due to a relatively small group of women with overweight/obesity and GWG below the IOM recommendations, our results must be interpreted with caution.

The main strengths of this study include a large, well-defined clinical and homogenous study cohort (99.3% of women with the Finnish ancestry). The GDM status of all participants was confirmed from their medical records, and several potential confounders were considered. We were also able to stratify our analyses by pre-pregnancy BMI. The study provides high-quality reference data of GWG in a relation with GDM status and fetal overgrowth. There were also some limitations in this study. Due to the low number of underweight women (*n* = 59, 2.8%), they were categorized within the normal weight group. Thus, the additional analysis was made after excluding underweight women and findings did not change. Also, the number of women with GWG below the recommendations was limited especially those with overweight or obesity. Therefore, these women were classified as having normal GWG in the final analysis. However, the results were congruent regardless of whether the women with GWG below recommendations were included in the normal GWG group or observed separately. Another limitation was the small number of non-diabetic women with obesity, and especially, with normal GWG. Probably therefore, in this group, the effect of normal GWG remained statistically non-significant. In addition, pre-pregnancy weight was self-reported during the first antenatal visit. This is common in similar studies, and as mothers are measured at the first antenatal visit, substantial reporting error is unlikely. Also the timing of GWG has been reported relevant to fetal growth (18–20). Though excessive GWG early in pregnancy has been reported to have the strongest effect on fetal overgrowth (18, 19), also controversial results has been presented (20). Thus, another limitation of the study is that we were able to study only the total GWG during pregnancy, not weight gain in different trimesters of pregnancy or after GDM diagnosis. Although ethnically homogenous study population is the strength of the study, the results may not be universally applicable to other ethnic groups.

The diagnostic criteria still vary across the globe. In the FinnGeDi study, the diagnosis of GDM was based on Finnish Current Care Guidelines published in 2008 and revised in 2013 (14). This recommendation was primarily launched before currently widely used the International Association of Diabetes and Pregnancy Study Group (IADPSG) criteria were published in 2010 (21). Based on Hyperglycaemia and Adverse Pregnancy Outcome study (HAPO), the cut-offs for fasting glucose (≥ 5.1 mmol/l) and 2 h glucose (≥ 8.5 mmol/l) are slightly lower according to the IADPSG criteria. Thus, the prevalence of GDM is slightly lower according to the Finnish criteria. In this study population 62 women with fasting glucose 5.1–5.2 mmol/l would have been diagnosed with GDM according to the IADPSG criteria. No additional cases of GDM would have been found based on 2 h value of 8.5 mmol/l. Thus, according to the Finnish guidelines, only a small group of women was not diagnosed with GDM and hence was not treated. The diagnostic criteria used in this study, were and still are generally applied in the whole Finland.

Normal GWG seems to decrease the risk for LGA especially in GDM women with pre-pregnancy obesity and in non-diabetic women with normal weight. Among both GDM and non-diabetic women with normal weight, birth weight SD scores of the newborns were lower when GWG was in the normal range. Regardless of maternal glycemic status, effective prevention of excess GWG, especially in women with obesity, is essential to reduce fetal overgrowth.

## Data Availability Statement

The datasets presented in this article are not readily available because data cannot be shared for both legal and ethical reasons. Data from the Finnish Institute for Health and Welfare can only be used for the purpose stated in the license granted, scientific research on society by the license applicant, and can therefore not be shared with third parties. Researchers can apply for data through the authorization application process at Finnish Institute for Health and Welfare. Requests to access the datasets should be directed to Sanna Mustaniemi, sanna.mustaniemi@oulu.fi.

## Ethics Statement

The studies involving human participants were reviewed and approved by the Ethics Committee in Northern Ostrobothnia Hospital District, Oulu, Finland. Number 2008/43, date of approval 19 June 2008. The participants provided their written informed consent to participate in this study.

## Author Contributions

MV, AP, HL, JE, MG, RK, and EK initiated and designed the FinnGeDi study. MV, HN, EK, and SM designed the present study. SM and AB performed data analysis. SM wrote the first draft of the manuscript, and HN, EK, and MV reviewed, completed, and supervised the manuscript writing. All authors contributed to its interpretation and contributed to the article and approved the submitted version.

## Conflict of Interest

The authors declare that the research was conducted in the absence of any commercial or financial relationships that could be construed as a potential conflict of interest.

## Abbreviations

GWG: gestational weight gain
GDM: gestational diabetes
LGA: large-for-gestational age
IOM: Institute of Medicine
OGTT: oral glucose tolerance test.

## References

[1] Guariguata L Linnenkamp U Beagley J Whiting DR Cho NH. Global estimates of the prevalence of hyperglycaemia in pregnancy. Diabetes Res Clin Pract. (2014) 103:176–85. 10.1016/j.diabres.2013.11.003 24300020

[2] Koivunen S Kajantie E Torkki A Bloigu A Gissler M Pouta A. The changing face of gestational diabetes: the effect of the shift from risk factor-based to comprehensive screening. Eur J Endocrinol. (2015) 173:623–32. 10.1530/EJE-15-0294 26282598

[3] Institute of Medicine (US) and National Research Council (US) Committee to Reexamine IOM Pregnancy Weight Guidelines. Washington, DC (2009).

[4] Goldstein RF Abell SK Ranasinha S Misso M Boyle JA Black MH. Association of gestational weight gain with maternal and infant outcomes: a systematic review and meta-analysis. J Am Med Assoc. (2017) 317:2207–25. 10.1001/jama.2017.3635 28586887 PMC5815056

[5] Kim SY Sharma AJ Sappenfield W Wilson HG Salihu HM. Association of maternal body mass index, excessive weight gain, and gestational diabetes mellitus with large-for-gestational-age births. Obstet Gynecol. (2014) 123:737–44. 10.1097/AOG.0000000000000177 24785599 PMC4548850

[6] Egan AM Dennedy MC Al-Ramli W Heerey A Avalos G Dunne F. ATLANTIC-DIP: excessive gestational weight gain and pregnancy outcomes in women with gestational or pregestational diabetes mellitus. J Clin Endocrinol Metab. (2014) 99:212–9. 10.1210/jc.2013-2684 24187402

[7] Lee JM Kim MJ Kim MY Han JY Ahn HK Choi JS. Gestational weight gain is an important risk factor for excessive fetal growth. Obstet Gynecol Sci. (2014) 57:442. 10.5468/ogs.2014.57.6.442 25469331 PMC4245336

[8] Black MH Sacks DA Xiang AH Lawrence JM. The relative contribution of prepregnancy overweight and obesity, gestational weight gain, and IADPSG-defined gestational diabetes mellitus to fetal overgrowth. Diabetes Care. (2013) 36:56–62. 10.2337/dc12-0741 22891256 PMC3526206

[9] Ouzounian JG Hernandez GD Korst LM Montoro MM Battista LR Walden CL. Pre-pregnancy weight and excess weight gain are risk factors for macrosomia in women with gestational diabetes. J Perinatol. (2011) 31:717–21. 10.1038/jp.2011.15 21372797

[10] Chen Q Wei J Tong M Yu L Lee AC Gao YF. Associations between body mass index and maternal weight gain on the delivery of LGA infants in Chinese women with gestational diabetes mellitus. J Diabetes Complications. (2015) 29:1037–41. 10.1016/j.jdiacomp.2015.08.017 26376766

[11] Wong T Barnes RA Ross GP Cheung NW Flack JR. Are the Institute of Medicine weight gain targets applicable in women with gestational diabetes mellitus? Diabetologia. (2017) 60:416–23. 10.1007/s00125-016-4173-3 27942798

[12] Mustaniemi S Vääräsmäki M Eriksson JG Gissler M Laivuori H Ijäs H. Polycystic ovary syndrome and risk factors for gestational diabetes. Endocr Connect. (2018) 7:859–69. 10.1530/EC-18-0076 29858213 PMC6026881

[13] Keikkala E Mustaniemi S Koivunen S Kinnunen J Viljakainen M Männisto T. Cohort profile: the finnish gestational diabetes (FinnGeDi) study. Int J Epidemiol. (2020) 49:1–9. 10.1093/ije/dyaa039 32374401 PMC7394962

[14] Finnish Medical Society Duodecim. Current Care Guidelines for Gestational Diabetes. Helsinki: The Finnish Medical Society Duodecim (2008). Available online at: www.kaypahoito.fi (accessed March 3, 2014).

[15] Obesity: preventing and managing the global epidemic. Report of a WHO consultation. World Health Organ Tech Rep Ser. (2000) 894:i–xii, 1–253. 11234459

[16] Sankilampi U Hannila ML Saari A Gissler M Dunkel L. New population-based references for birth weight, length, and head circumference in singletons and twins from 23 to 43 gestation weeks. Ann Med. (2013) 45:446–54. 10.3109/07853890.2013.803739 23768051

[17] Goldstein RF Abell SK Ranasinha S Misso ML Boyle JA Harrison CL. Gestational weight gain across continents and ethnicity: systematic review and meta-analysis of maternal and infant outcomes in more than one million women. BMC Med. (2018) 16:1–14. 10.1186/s12916-018-1128-1 30165842 PMC6117916

[18] Retnakaran R Wen SW Tan H Zhou S Ye C Shen M. Association of timing of weight gain in pregnancy with infant birth weight. JAMA Pediatr. (2018) 172:136–42. 10.1001/jamapediatrics.2017.4016 29279903 PMC5796742

[19] Broskey NT Wang P Li N Leng J Li W Wang L. Early pregnancy weight gain exerts the strongest effect on birth weight, posing a critical time to prevent childhood obesity. Obesity. (2017) 25:1569–76. 10.1002/oby.21878 28845614 PMC5604854

[20] Wu Y Wan S Gu S Mou Z Dong L Luo Z. Gestational weight gain and adverse pregnancy outcomes: a prospective cohort study. BMJ Open. (2020) 10:1–8. 10.1136/bmjopen-2020-038187 32878761 PMC7470642

[21] International Association of Diabetes Pregnancy Study Groups Consensus Panel Metzger BE Gabbe SG Persson B Buchanan TA Catalano PA . International association of diabetes and pregnancy study groups recommendations on the diagnosis and classification of hyperglycemia in pregnancy. Diabetes Care. (2010) 33:676–82. 10.2337/dc09-1848 20190296 PMC2827530

